# Comparative Evaluation of Three Probiotics for Streptococcus mutans Prevention in Plaque Around Orthodontic Braces

**DOI:** 10.7759/cureus.37923

**Published:** 2023-04-21

**Authors:** Shalabh Baxi, Virag Bhatia, Anand A Tripathi, Pratiksha Kumar, Anurag Tiwari, Hiroj Bagde

**Affiliations:** 1 Department of Orthodontics, Government Dental College, Raipur, IND; 2 Department of Orthodontics, Government College of Dentistry, Indore, IND; 3 Department of Orthodontics, Saraswati-Dhanwantari Dental College and Hospital, Parbhani, IND; 4 Department of Oral Pathology and Microbiology, Government College of Dentistry, Indore, IND; 5 Department of Orthodontics and Dentofacial Orthopedics, Saraswati-Dhanwantari Dental College and Hospital, Parbhani, IND; 6 Department of Periodontology, Rama Dental College and Research Centre, Kanpur, IND

**Keywords:** s. mutans, colonies, plaque, orthodontic appliances, probiotics

## Abstract

Background and aim: Orthodontic brackets can be a significant factor in enamel demineralization due to their complex structure, which makes brushing the teeth difficult and promotes the accumulation of food particles and dental plaque. The fact that metal braces have the highest surface tension and are more likely to cause enamel demineralization, which can result in the development of white spot lesions and enamel caries, is of critical significance to doctors, dentists, and patients. Probiotics have a beneficial effect in preventing and treating oral infectious diseases like tooth decay, gingival disorders, and bad breath. Research has shown that taking probiotics lowers the amount of *Streptococcus mutans *in the body. There has not been much research done to examine the results of administering a probiotic medication locally. This study was conducted to examine the effectiveness of three separate probiotics in the prevention of *S. mutans *accumulation in plaque surrounding orthodontic braces.

Materials and methods: A randomized controlled trial was conducted. The volunteers for each group were chosen using a straightforward random method. The sample size was 160 empirically determined subjects. They were divided as follows: study group 1 received probiotic lozenges (n=40). Study group 2 received probiotic sachets (n=40). Study group 3 received probiotic beverages (n=40). Group 4 was the control group, those who did not get probiotics (n=40). The samples were then plated onto culture media to test for *Streptococcus mutans*. *S. mutans *colonies were counted using a computerized colony counter.

Results: The mean values of colony forming units (CFU/mL) of *S. mutans *at baseline in the control group were 354±23.6, while they were 232±41.7 at the end of the observation duration. The difference was non-relevant statistically (p=0.793). The mean values of CFU/mL of *S. mutans* at baseline in the group taking probiotic lozenges were 358.7±39.93, while they were 57±10.12 at the end of the observation duration. The difference was relevant statistically (p=0.021). The mean values of CFU/mL of *S. mutans *at baseline in the group taking probiotic sachets were 321.36±41.67, while they were 215.5±22.66 at the end of the observation duration. The difference was relevant statistically (p=0.043). The mean values of CFU/mL of *S. mutans *at baseline in the group taking the probiotic drink were 335.76±40.12, while they were 75.1±28.74 at the end of the observation duration. The difference was relevant statistically (p=0.032).

Conclusion: There was a significant decline in the number of colonies of *S. mutans *in all three forms of probiotics; however, the decline was greatest in the study participants taking probiotic lozenges.

## Introduction

Orthodontics is the branch of dentistry concerned with the prevention, detection, and treatment of malocclusions and other irregularities of the dentofacial system. In current history, orthodontic treatment has become a vital component of dental care, and this pattern is predicted to persist in the coming times. Long-term consequences of orthodontic therapy showed that most of those who underwent it felt it was beneficial and were pleased with the outcome [1]. Rehabilitation with fixed orthodontic appliances may be connected with gingivitis and enamel demineralization, despite the fact that many individuals report remarkable improvements in their tooth and facial aesthetics, occlusal functioning, and dental health [1,2].

While enamel demineralization is typically irreversible, gingivitis can be treated. Despite improvements in treatment methods and appliance cleanliness, plaque deposition still occurs in certain spots, particularly near orthodontic brackets, orthodontic bands, orthodontic wires, and other connectors. It produces particular alterations in the saliva, such as a drop in pH, which boosts the formation of infectious plaque and demineralizes enamel. Individuals undergoing orthodontic therapy have greater concentrations of acid-generating anaerobic microorganisms in their plaque than those without orthodontic devices, especially *Streptococcus mutans* which has been identified as the primary cause of tooth caries [3-6]. Among various orthodontic devices, orthodontic brackets can be a significant factor in enamel demineralization due to their complex structure, which makes brushing the teeth difficult and promotes the accumulation of food particles and dental plaque. The fact that metal braces have the highest surface tension and are more likely to cause enamel demineralization, which can result in the development of white spot lesions and enamel caries, is of critical significance to doctors, dentists, and patients [7-9].

Probiotics are growing in interest as an intriguing subject in the modern antibiotic-driven sector. Regularization of the gut microbiota, immune system response regulation, and metabolic action might be considered the three main probiotic fundamental processes. Probiotics may prevent the development of white spot defects caused by the decalcification of their tooth enamel in orthodontic patients [10,11].

Although only a few studies have been conducted to date, the outcomes of these investigations have indicated the positive impact of probiotics in preventing and managing oral infectious diseases, such as tooth decay, gingival disorders, and bad breath. Research has shown that taking probiotics lowers the amount of *S. mutans *in the body [12,13]. There has not been much research done to examine the results of administering a probiotic medication locally. Therefore, we conducted this study to examine the effectiveness of three separate probiotics in the prevention of *S. mutans *accumulation in plaque surrounding orthodontic braces.

## Materials and methods

Data source

Based on the inclusion and exclusion criteria, the participants for the current research were selected from individuals receiving fixed appliance mechanotherapy for orthodontic treatment. Ethical approval for the study was obtained from the institute with the institutional review board number 02/IEC/RDCHRC/2022-23/042.

**Figure 1:**
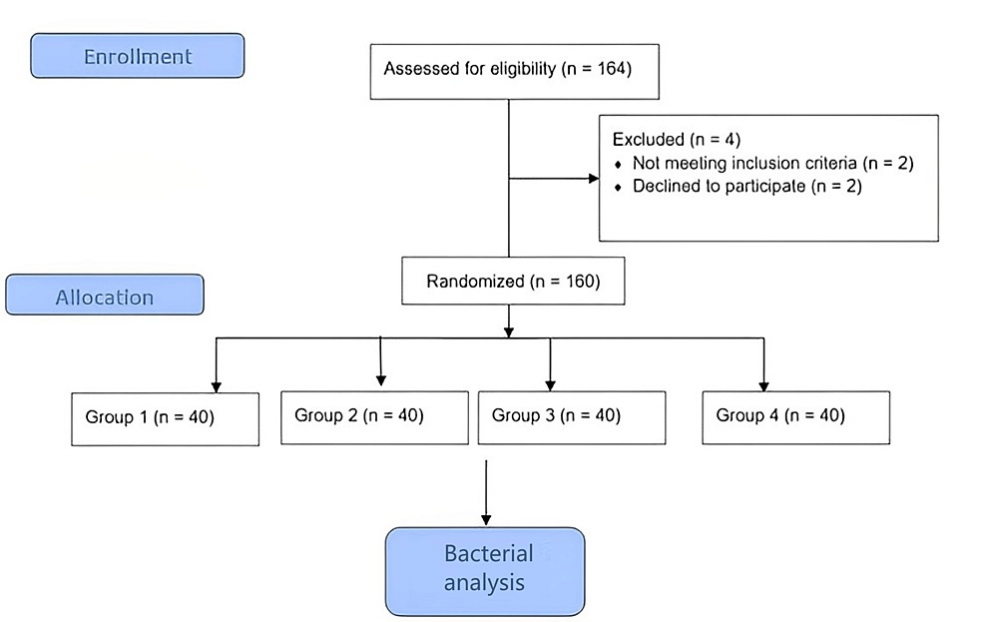
CONSORT flow for the study. CONSORT: Consolidated Standards of Reporting Trials

A randomized controlled trial was conducted. Volunteers for each group were chosen using a straightforward random method by use of a computer-generated list. A 160-person sample size was present for the study out of the 164 accessed, where two declined to participate and there were no dropouts (Figure 1).

Study groups

Study group 1 received probiotic lozenges (n=40); study group 2 received probiotic sachets (n=40); study group 3 received probiotic beverages (n=40); and study group 4 was the control group, those who did not receive probiotics (n=40).

Patient selection

Inclusion criteria include patients receiving orthodontic treatment for at least nine months and up to 12 months, both genders between the ages of 14 and 29 years, permanent dentition in the patient, patients with good overall health, and patients with signed informed consent forms and have given their permission voluntarily. Exclusion criteria include patients having an extensive medical history of immunological diseases and persistent systemic sickness, patients taking any other medications during the study or in the month before, patients who received topical fluoride therapy within the previous four weeks, patients with untreated active caries, gingival irritation, or poor oral health, and patients having a history of using mouthwash or chewing gum both before and throughout the study.

Criteria for elimination from the study

Those who participated in the research and would have been referred to a general practitioner were removed from the study, and if they had any medical conditions that would have necessitated antibiotic treatment over the course of the study were also excluded.

Collection of samples

The same operator gently removed the elastomeric modules at each interval to release the archwires. Plaque samples were obtained using the four-pass approach recommended by Pellegrini et al. from the labial surfaces of the maxillary lateral incisors that were immediately adjacent to the orthodontic brackets [7]. In order to avoid overloading the tooltip, four passes were made along the tooth at the gingival, mesial, distal, and occlusal aspects of the bracket contact.

Inoculation of samples

Plaque samples were collected in sterile, 10% thioglycollate broth-filled screw-cap vials that served as a transit medium. The samples were then plated onto culture media to test for *Streptococcus mutans*. *S. mutans *colonies were counted using a recognized colony counter.

Statistical analysis

SPSS software version 21 (Armonk, NY: IBM Corp.) was used to compile and analyze the data using the correct statistical methodology. In order to determine the statistical significance of the findings, data comparison was carried out using certain statistical tests like paired t-test and ANOVA. At baseline (0 days) and 30 days into the intervention, the mean and standard deviation of *S. mutans *CFU were determined. *S. mutans *CFU were compared pairwise before and after probiotic formulation administration in study groups and without probiotic use in the control group using the paired t-test. Using one-way analysis of variance (ANOVA), followed by Tukey's post hoc test, the variation of *S. mutans *CFU between various groups at baseline and the variation of *S. mutans *CFU between different groups after 30 days were analyzed. P-values under 0.05 were considered statistically significant.

## Results

The mean values of CFU/mL of *S. mutans *at baseline in the control group were 354±23.6, while they were 232±41.7 at the end of the observation duration. The difference was non-relevant statistically (p=0.793). The mean values of CFU/mL of *S. mutans *at baseline in the group taking probiotic lozenges were 358.7±39.93, while they were 57±10.12 at the end of the observation duration. The difference was relevant statistically (p=0.021). The mean values of CFU/mL of *S. mutans *at baseline in the group taking probiotic sachets were 321.36±41.67, while they were 215.5±22.66 at the end of the observation duration. The difference was relevant statistically (p=0.043). The mean values of CFU/mL of *S. mutans *at baseline in the group taking probiotic drinks were 335.76±40.12, while they were 75.1±28.74 at the end of the observation duration. The difference was relevant statistically (p=0.032) (Table 1).

**Table 1:** Comparison of the mean values of CFU/mL of S. mutans.

Groups	Control	Probiotic lozenges	Probiotic sachet	Probiotic drink
Baseline CFU/mL (10^3^) mean±SD	354±23.6	358.7±39.93	321.36±41.67	335.76±40.12
After CFU/mL (10^3^) mean±SD	232±41.7	57±10.12	215.5±22.66	75.1±28.74
t-test	0.527	2.963	2.428	2.625
P-value	0.793	0.021	0.043	0.032

It was observed that there was a significant decline in the number of colonies of *S. mutans *in all three forms of probiotics; however, the decline was greatest in study participants taking probiotic lozenges. The mean values of CFU/mL of *S. mutans *at baseline in control group was 354±23.6, while it was 232±41.7 at the end of the observation duration. The difference was non-relevant statistically (p=0.793). The mean values of CFU/mL of *S. mutans *at baseline in group taking probiotic lozenges was 358.7±39.93, while it was 57±10.12 at the end of the observation duration. The difference was relevant statistically (p=0.021). The mean values of CFU/mL of *S. mutans* at baseline in group taking probiotic sachets was 321.36±41.67, while it was 215.5±22.66 at the end of observation duration. The difference was relevant statistically (p=0.043). The mean values of CFU/mL of *S. mutans *at baseline in group taking probiotic drink was 335.76±40.12, while it was 75.1±28.74 at the end of the observation duration. The difference was relevant statistically (p=0.032) (Table 1). It was observed that there was a significant decline in the number of colonies of *S. mutans *in all three forms of probiotics; however, the decline was maximum in study participants taking probiotic lozenges (Table 2).

**Table 2:** Comparison of S. mutans CFU between different groups at baseline and the variation of S. mutans CFU within groups after 30 days. DF: degree of freedom

Variables	Baseline CFU			After CFU		
	Between groups	Within groups	Total	Between groups	Within groups	Total
Sum of squares	1636.432	127373.249	1290096.732	13164.444	333036.878	464680.111
Df	3	76	79	3	76	79
Mean square	5454.821	1675.981	-	43881.221	43820.738	-
F	0.326			10.015		-
P-value	0.818			0.003		-
Tukey's post hoc test	1=2=3=4			1>3>2>4		

## Discussion

Probiotics are becoming an increasingly fascinating topic in the modern antibiotic-driven industry. The three primary probiotic fundamental mechanisms might be metabolic action, immune system response modulation, and regulation of ions in the gut flora. Orthodontic patients may benefit from probiotics to prevent the development of white spot problems brought on by tooth enamel decalcification. In particular, *Streptococcus mutans*, recognized as the main cause of dental caries, is more prevalent in the plaque of people receiving orthodontic therapy than in the plaque of people not receiving it [14,15].

Due to the difficulty of brushing teeth caused by the complicated construction of orthodontic brackets, which promotes the buildup of food debris and dental plaque, orthodontic brackets can be a substantial contributor to enamel demineralization. The fact that metal braces have the highest surface tension and are more likely to demineralize enamel, which can lead to the emergence of white spot lesions and enamel caries, is critically important for physicians, dentists, and patients [16,17]. Even though few studies have been performed to date, the findings show that probiotics are effective in preventing and treating oral infectious diseases, such as tooth decay, gingival problems, and bad breath. According to research, consuming probiotics reduces the *S. mutans *population in the body [18]. Few studies have been conducted to evaluate the outcomes of giving probiotic medicine locally. In order to determine how well three different probiotics inhibit the bacteria *S. mutans *from growing in plaque around orthodontic braces, we conducted this study.

Our research observed that the mean values of CFU/mL of *S. mutans *at baseline in the control group were 354±23.6, while they were 232±41.7 at the end of the observation duration. The difference was non-relevant statistically (p=0.793). The mean values of CFU/mL of *S. mutans *at baseline in the group taking probiotic lozenges were 358.7±39.93, while they were 57±10.12 at the end of the observation duration. The difference was relevant statistically (p=0.021). The mean values of CFU/mL of *S. mutans *at baseline in the group taking probiotic sachets were 321.36±41.67, while they were 215.5±22.66 at the end of the observation duration. The difference was relevant statistically (p=0.043). The mean values of CFU/mL of *S. mutans *at baseline in the group taking probiotic drinks were 335.76±40.12, while they were 75.1±28.74 at the end of the observation duration. The difference was relevant statistically (p=0.032).

The prevention, identification, and treatment of malocclusions and other anomalies of the dentofacial system are the focus of the dental specialty known as orthodontics. In current history, orthodontic therapy has become a vital component of dental care, and this pattern is projected to continue in the future. A study on the long-term effects of orthodontic treatment found that the majority of those who received it thought it was beneficial and were pleased with the results. Although many people report significant gains in their teeth and visual esthetics, occlusal function, and dental health, rehabilitation with fixed appliances for orthodontics has disadvantages because it may be linked with gingivitis and enamel demineralization.

All three types of probiotics were shown to significantly reduce the number of *S. mutans *colonies in the participants of this study, but the drop was greatest in those receiving probiotic lozenges. Gingivitis can be treated; however, enamel demineralization is usually irreversible [19,20]. Plaque deposition still happens in some areas, especially close to orthodontic brackets, bands, wires, and other connectors, despite advancements in treatment techniques and appliance hygiene. It causes specific salivary changes, like a reduction in pH, which promotes the growth of infectious plaque and demineralizes enamel [12,17].

The study limitations are the small sample size and various other Gram-negative pathogenic species were not checked for their presence.

## Conclusions

Probiotics have been proven to have beneficial effects in reducing the ill effects of pathogenic bacteria. Number of colonies of various pathogenic bacteria can be regulated by the positive effects of the probiotics. In this study, there has been reported use of probiotics to have an effect on the pathogenic bacterial strains. There was a significant decline in the number of colonies of *S. mutans *in all three forms of probiotics; however, the decline was greatest in study participants taking probiotic lozenges.
